# Methyl *N*-(4-chlorophenyl)succinamate

**DOI:** 10.1107/S1600536809002724

**Published:** 2009-01-28

**Authors:** B. Thimme Gowda, Sabine Foro, B. S. Saraswathi, Hiromitsu Terao, Hartmut Fuess

**Affiliations:** a Department of Chemistry, Mangalore University, Mangalagangotri 574 199, Mangalore, India; b Institute of Materials Science, Darmstadt University of Technology, Petersenstrasse 23, D-64287 Darmstadt, Germany; c Faculty of Integrated Arts and Sciences, Tokushima University, Minamijosanjima-cho, Tokushima 770-8502, Japan

## Abstract

In the structure of the title compound {systematic name: methyl 3-[(4-chloro­phen­yl)amino­carbon­yl]propionate}, C_11_H_12_ClNO_3_, the conformations of the N—H and C=O bonds in the amide fragment are *trans* to each other and the conformations of the amide O atom and the carbonyl O atom of the ester fragment are also *trans* to the H atoms attached to the adjacent C atoms. Mol­ecules are linked into a centrosymmetric *R*_2_^2^(14) dimer by simple N—H⋯O inter­actions. Furthermore, a short intra­molecular C—H⋯O contact may stabilize the conformation adopted by the mol­ecule in the crystal.

## Related literature

For background, see: Gowda *et al.* (2007 ▶); Gowda, Foro & Fuess (2008 ▶); Gowda, Foro, Sowmya *et al.* (2008 ▶); Jones *et al.* (1990 ▶); Wan *et al.* (2006 ▶). For related literature, see: Bernstein *et al.* (1995 ▶).
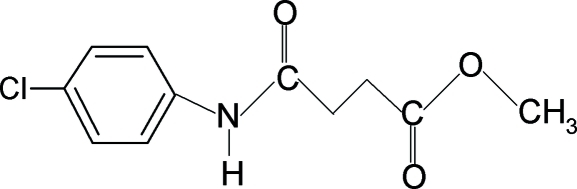


## Experimental

### 

#### Crystal data

C_11_H_12_ClNO_3_
*M* *_r_* = 241.67
Orthorhombic, 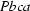

*a* = 14.190 (1) Å
*b* = 5.6370 (5) Å
*c* = 28.139 (3) Å
*V* = 2250.8 (4) Å^3^
*Z* = 8
Mo *K*α radiation
μ = 0.33 mm^−1^
*T* = 299 (2) K
0.50 × 0.48 × 0.44 mm

#### Data collection

Oxford Diffraction Xcalibur diffractometer with Sapphire CCD detector
Absorption correction: multi-scan (*CrysAlis RED*; Oxford Diffraction, 2007 ▶) *T*_min_ = 0.852, *T*_max_ = 0.868
10377 measured reflections
2272 independent reflections
1649 reflections with *I* > 2σ(*I*)
*R*_int_ = 0.043

#### Refinement

*R*[*F* ^2^ > 2σ(*F* ^2^)] = 0.050
*wR*(*F* ^2^) = 0.154
*S* = 1.19
2272 reflections
173 parameters
H atoms treated by a mixture of independent and constrained refinement
Δρ_max_ = 0.28 e Å^−3^
Δρ_min_ = −0.27 e Å^−3^

### 

Data collection: *CrysAlis CCD* (Oxford Diffraction, 2004 ▶); cell refinement: *CrysAlis RED* (Oxford Diffraction, 2007 ▶); data reduction: *CrysAlis RED*; program(s) used to solve structure: *SHELXS97* (Sheldrick, 2008 ▶); program(s) used to refine structure: *SHELXL97* (Sheldrick, 2008 ▶); molecular graphics: *PLATON* (Spek, 2003 ▶); software used to prepare material for publication: *SHELXL97*.

## Supplementary Material

Crystal structure: contains datablocks I, global. DOI: 10.1107/S1600536809002724/bx2194sup1.cif

Structure factors: contains datablocks I. DOI: 10.1107/S1600536809002724/bx2194Isup2.hkl

Additional supplementary materials:  crystallographic information; 3D view; checkCIF report

## Figures and Tables

**Table 1:** Hydrogen-bond geometry (Å, °)

*D*—H⋯*A*	*D*—H	H⋯*A*	*D*⋯*A*	*D*—H⋯*A*
C6—H6⋯O1	0.94 (3)	2.22 (3)	2.833 (4)	121 (3)
N1—H1*N*⋯O2^i^	0.82 (3)	2.22 (3)	3.020 (3)	163 (3)

Symmetry code: (i) 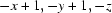.

**Fig. 1.**
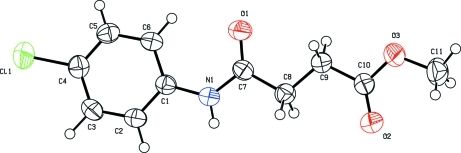
Molecular structure of the title compound, showing the atom labeling scheme. The displacement ellipsoids are drawn at the 50% probability level. The H atoms are represented as small spheres of arbitrary radii.

**Fig. 2.**
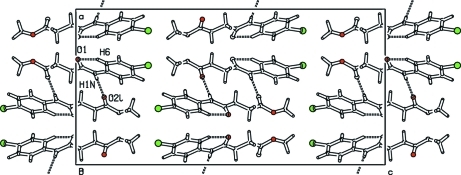
A fragment of the structure of (I) , viewed along the b axis , dashed lines. shown N-H···O and C-H···O interactions

## supplementary crystallographic information

### Comment

Amides are of interest as conjugation between the nitrogen lone pair electrons and the carbonyl pi-bond results in distinct physical and chemical properties. The amide moiety is also an important constituent of many biologically significant compounds. Thus, the structural studies of amides are of interest (see Gowda *et al.*, 2007 and references therein; Gowda, Foro & Fuess, 2008; Gowda, Foro, Sowmya *et al.*, 2008; Jones *et al.*, 1990; Wan *et al.*, 2006 as representative examples). As a part of studying the effect of ring and side-chain substitutions on the solid state geometry of this class of compounds, we report herein the crystal structure of *N*-(4-chlorophenyl)methylsuccinamate (N4CPMSA). The conformations of N—H and C=O bonds in the amide fragment are *trans* to each other and the conformations of the amide oxygen and the carbonyl oxygen of the ester segment are also *trans* to the H-atoms attached to the adjacent carbons (Fig. 1). The succinamido group and the benzene ring lie in the same plane with the Rms deviation of fitted atoms equal to 0.0720 Å. The molecules are linked into centrosymmetric R~2~^2^(14) dimer by simple N-H···O interactions (Bernstein *et al.*,  1995). Furthermore, a short intramolecular C-H···.O contact may stabilize the conformation adopted by the molecule in the solid state (Table 1) is shown in Fig.2.

### Experimental

The solution of succinic anhydride (0.025 mole) in toluene (25 cc) was treated dropwise with the solution of 4-chloroaniline (0.025 mole) in toluene (20 cc) with constant stirring. The resulting mixture was stirred for about one hour and set aside for an additional hour at room temperature for completion of the reaction. The mixture was then treated with dilute hydrochloric acid to remove the unreacted 4-chloroaniline. The resultant solid *N*-(4-chlorophenyl)-succinamic acid was filtered under suction and washed thoroughly with water to remove the unreacted succinic anhydride and succinic acid. The slow crystallization of *N*-(4-chlorophenyl)-succinamic acid in hot methanol resulted in *N*-(4-chlorophenyl)-methylsuccinamate. It was further recrystallized to constant melting point from methanol. The purity of the compound was checked by elemental analysis and characterized by recording its infrared and NMR spectra. The single crystals used in X-ray diffraction studies were grown in methanolic solution by slow evaporation at room temperature.

### Refinement

The H atoms of the methyl group were positioned with idealized geometry using a riding model with C—H = 0.96 Å. The other H atoms were located in difference map, and their positional parameters were refined freely [N—H = 0.82 (3) Å, C—H = 0.90 (3)–1.01 (3) Å]. All H atoms were refined with isotropic displacement parameters with U_iso_(H) = 1.2 U_eq_(C-aromatic,N) or 1.5 U_eq_ (C-methyl).

### Crystal data

**Table:**

C_11_H_12_ClNO_3_	*F*(000) = 1008
*M**_r_* = 241.67	*D*_x_ = 1.426 Mg m^−^^3^
Orthorhombic, *P**b**c**a*	Mo *K*α radiation, λ = 0.71073 Å
Hall symbol: -P 2ac 2ab	Cell parameters from 3422 reflections
*a* = 14.190 (1) Å	θ = 2.6–28.0°
*b* = 5.6370 (5) Å	µ = 0.33 mm^−^^1^
*c* = 28.139 (3) Å	*T* = 299 K
*V* = 2250.8 (4) Å^3^	Prism, colourless
*Z* = 8	0.50 × 0.48 × 0.44 mm

### Data collection

**Table:**

Oxford Diffraction Xcalibur diffractometer with Sapphire CCD detector	2272 independent reflections
Radiation source: fine-focus sealed tube	1649 reflections with *I* > 2σ(*I*)
graphite	*R*_int_ = 0.043
Rotation method data acquisition using ω and φ scans	θ_max_ = 26.4°, θ_min_ = 2.9°
Absorption correction: multi-scan (*CrysAlis RED*; Oxford Diffraction, 2007)	*h* = −17→17
*T*_min_ = 0.852, *T*_max_ = 0.868	*k* = −7→7
10377 measured reflections	*l* = −33→35

### Refinement

**Table:**

Refinement on *F*^2^	Secondary atom site location: difference Fourier map
Least-squares matrix: full	Hydrogen site location: inferred from neighbouring sites
*R*[*F*^2^ > 2σ(*F*^2^)] = 0.050	H atoms treated by a mixture of independent and constrained refinement
*wR*(*F*^2^) = 0.154	*w* = 1/[σ^2^(*F*_o_^2^) + (0.0501*P*)^2^ + 2.2683*P*] where *P* = (*F*_o_^2^ + 2*F*_c_^2^)/3
*S* = 1.19	(Δ/σ)_max_ < 0.001
2272 reflections	Δρ_max_ = 0.28 e Å^−^^3^
173 parameters	Δρ_min_ = −0.27 e Å^−^^3^
0 restraints	Extinction correction: *SHELXL97* (Sheldrick, 2008), Fc^*^=kFc[1+0.001xFc^2^λ^3^/sin(2θ)]^-1/4^
Primary atom site location: structure-invariant direct methods	Extinction coefficient: 0.0109 (13)

### Special details

**Table:**

Geometry. All e.s.d.'s (except the e.s.d. in the dihedral angle between two l.s. planes) are estimated using the full covariance matrix. The cell e.s.d.'s are taken into account individually in the estimation of e.s.d.'s in distances, angles and torsion angles; correlations between e.s.d.'s in cell parameters are only used when they are defined by crystal symmetry. An approximate (isotropic) treatment of cell e.s.d.'s is used for estimating e.s.d.'s involving l.s. planes.
Refinement. Refinement of *F*^2^ against ALL reflections. The weighted *R*-factor *wR* and goodness of fit *S* are based on *F*^2^, conventional *R*-factors *R* are based on *F*, with *F* set to zero for negative *F*^2^. The threshold expression of *F*^2^ > σ(*F*^2^) is used only for calculating *R*-factors(gt) *etc*. and is not relevant to the choice of reflections for refinement. *R*-factors based on *F*^2^ are statistically about twice as large as those based on *F*, and *R*- factors based on ALL data will be even larger.

### Fractional atomic coordinates and isotropic or equivalent isotropic displacement parameters (Å^2^)

**Table:**

	*x*	*y*	*z*	*U*_iso_*/*U*_eq_	
Cl1	0.63844 (7)	1.51417 (17)	0.23361 (3)	0.0671 (3)	
O1	0.67752 (18)	1.1013 (4)	0.00684 (8)	0.0659 (7)	
O2	0.56516 (16)	0.4234 (4)	−0.09121 (8)	0.0591 (6)	
O3	0.66393 (16)	0.6160 (5)	−0.13895 (8)	0.0635 (7)	
N1	0.58842 (17)	0.9188 (5)	0.06256 (8)	0.0436 (6)	
H1N	0.554 (2)	0.804 (6)	0.0675 (12)	0.052*	
C1	0.60124 (18)	1.0708 (5)	0.10151 (9)	0.0384 (6)	
C2	0.5627 (2)	1.0015 (6)	0.14469 (11)	0.0485 (7)	
H2	0.525 (2)	0.866 (6)	0.1470 (11)	0.058*	
C3	0.5731 (2)	1.1346 (6)	0.18498 (11)	0.0526 (8)	
H3	0.546 (2)	1.083 (6)	0.2168 (12)	0.063*	
C4	0.6234 (2)	1.3447 (5)	0.18267 (10)	0.0444 (7)	
C5	0.6611 (2)	1.4180 (5)	0.14030 (11)	0.0439 (7)	
H5	0.693 (2)	1.555 (6)	0.1384 (11)	0.053*	
C6	0.65037 (19)	1.2848 (5)	0.09955 (11)	0.0423 (6)	
H6	0.677 (2)	1.336 (6)	0.0706 (11)	0.051*	
C7	0.62722 (19)	0.9364 (5)	0.01857 (10)	0.0399 (6)	
C8	0.6017 (2)	0.7370 (5)	−0.01474 (10)	0.0418 (7)	
H8A	0.532 (2)	0.724 (5)	−0.0177 (10)	0.050*	
H8B	0.621 (2)	0.584 (6)	−0.0004 (11)	0.050*	
C9	0.6451 (2)	0.7706 (6)	−0.06280 (10)	0.0448 (7)	
H9A	0.712 (2)	0.773 (6)	−0.0601 (11)	0.054*	
H9B	0.628 (2)	0.926 (6)	−0.0768 (11)	0.054*	
C10	0.61912 (19)	0.5841 (5)	−0.09780 (10)	0.0417 (6)	
C11	0.6445 (3)	0.4460 (7)	−0.17585 (12)	0.0674 (10)	
H11A	0.6541	0.2885	−0.1638	0.081*	
H11B	0.6861	0.4731	−0.2022	0.081*	
H11C	0.5804	0.4629	−0.1862	0.081*	

### Atomic displacement parameters (Å^2^)

**Table:**

	*U*^11^	*U*^22^	*U*^33^	*U*^12^	*U*^13^	*U*^23^
Cl1	0.0874 (7)	0.0637 (6)	0.0502 (5)	−0.0054 (5)	0.0006 (4)	−0.0139 (4)
O1	0.0876 (17)	0.0558 (13)	0.0542 (13)	−0.0273 (13)	0.0239 (12)	−0.0081 (11)
O2	0.0642 (14)	0.0613 (14)	0.0520 (13)	−0.0215 (12)	0.0051 (10)	−0.0043 (11)
O3	0.0656 (14)	0.0797 (16)	0.0453 (12)	−0.0206 (13)	0.0149 (10)	−0.0109 (12)
N1	0.0475 (13)	0.0430 (14)	0.0403 (13)	−0.0096 (11)	0.0041 (10)	0.0028 (11)
C1	0.0374 (13)	0.0383 (14)	0.0396 (14)	0.0019 (11)	−0.0017 (11)	0.0051 (11)
C2	0.0546 (17)	0.0442 (16)	0.0467 (16)	−0.0106 (14)	0.0081 (13)	0.0033 (14)
C3	0.0629 (19)	0.0551 (19)	0.0398 (15)	−0.0065 (15)	0.0114 (14)	0.0034 (14)
C4	0.0473 (15)	0.0424 (16)	0.0436 (15)	0.0063 (13)	−0.0007 (12)	−0.0023 (12)
C5	0.0423 (15)	0.0366 (15)	0.0527 (17)	−0.0003 (12)	0.0028 (13)	−0.0003 (13)
C6	0.0444 (14)	0.0382 (15)	0.0442 (15)	−0.0025 (12)	0.0031 (12)	0.0071 (12)
C7	0.0392 (14)	0.0404 (15)	0.0401 (14)	0.0030 (12)	0.0031 (11)	0.0032 (12)
C8	0.0409 (14)	0.0426 (16)	0.0419 (15)	−0.0018 (13)	−0.0015 (12)	0.0014 (12)
C9	0.0440 (15)	0.0489 (17)	0.0415 (15)	−0.0067 (13)	0.0010 (12)	−0.0011 (13)
C10	0.0386 (14)	0.0468 (16)	0.0396 (14)	0.0019 (13)	−0.0003 (11)	0.0000 (12)
C11	0.068 (2)	0.085 (3)	0.0490 (18)	−0.007 (2)	0.0084 (16)	−0.0178 (18)

### Geometric parameters (Å, °)

**Table:**

Cl1—C4	1.736 (3)	C4—C5	1.371 (4)
O1—C7	1.217 (3)	C5—C6	1.379 (4)
O2—C10	1.200 (3)	C5—H5	0.90 (3)
O3—C10	1.333 (3)	C6—H6	0.94 (3)
O3—C11	1.440 (4)	C7—C8	1.507 (4)
N1—C7	1.358 (4)	C8—C9	1.498 (4)
N1—C1	1.403 (4)	C8—H8A	1.00 (3)
N1—H1N	0.82 (3)	C8—H8B	0.99 (3)
C1—C2	1.388 (4)	C9—C10	1.487 (4)
C1—C6	1.395 (4)	C9—H9A	0.95 (3)
C2—C3	1.367 (4)	C9—H9B	0.99 (3)
C2—H2	0.94 (3)	C11—H11A	0.9600
C3—C4	1.384 (4)	C11—H11B	0.9600
C3—H3	1.01 (3)	C11—H11C	0.9600
			
C10—O3—C11	116.4 (3)	O1—C7—C8	122.8 (3)
C7—N1—C1	127.9 (2)	N1—C7—C8	114.5 (2)
C7—N1—H1N	117 (2)	C9—C8—C7	111.6 (2)
C1—N1—H1N	115 (2)	C9—C8—H8A	110.0 (17)
C2—C1—C6	118.3 (3)	C7—C8—H8A	110.2 (17)
C2—C1—N1	117.4 (3)	C9—C8—H8B	111.4 (18)
C6—C1—N1	124.2 (2)	C7—C8—H8B	109.3 (18)
C3—C2—C1	121.9 (3)	H8A—C8—H8B	104 (2)
C3—C2—H2	117 (2)	C10—C9—C8	114.0 (2)
C1—C2—H2	121 (2)	C10—C9—H9A	108.0 (19)
C2—C3—C4	119.1 (3)	C8—C9—H9A	109.9 (19)
C2—C3—H3	122.3 (19)	C10—C9—H9B	107.4 (19)
C4—C3—H3	118.6 (19)	C8—C9—H9B	111.6 (19)
C5—C4—C3	120.0 (3)	H9A—C9—H9B	106 (3)
C5—C4—Cl1	120.3 (2)	O2—C10—O3	122.7 (3)
C3—C4—Cl1	119.7 (2)	O2—C10—C9	126.1 (3)
C4—C5—C6	121.0 (3)	O3—C10—C9	111.2 (2)
C4—C5—H5	121 (2)	O3—C11—H11A	109.5
C6—C5—H5	118 (2)	O3—C11—H11B	109.5
C5—C6—C1	119.6 (3)	H11A—C11—H11B	109.5
C5—C6—H6	120.5 (19)	O3—C11—H11C	109.5
C1—C6—H6	119.9 (19)	H11A—C11—H11C	109.5
O1—C7—N1	122.7 (3)	H11B—C11—H11C	109.5
			
C7—N1—C1—C2	−172.7 (3)	N1—C1—C6—C5	−178.2 (3)
C7—N1—C1—C6	6.9 (4)	C1—N1—C7—O1	−3.2 (5)
C6—C1—C2—C3	−1.1 (5)	C1—N1—C7—C8	177.7 (3)
N1—C1—C2—C3	178.6 (3)	O1—C7—C8—C9	−0.4 (4)
C1—C2—C3—C4	0.0 (5)	N1—C7—C8—C9	178.7 (3)
C2—C3—C4—C5	0.7 (5)	C7—C8—C9—C10	−177.6 (2)
C2—C3—C4—Cl1	−179.2 (3)	C11—O3—C10—O2	−0.1 (4)
C3—C4—C5—C6	−0.4 (4)	C11—O3—C10—C9	−180.0 (3)
Cl1—C4—C5—C6	179.6 (2)	C8—C9—C10—O2	3.6 (4)
C4—C5—C6—C1	−0.7 (4)	C8—C9—C10—O3	−176.5 (3)
C2—C1—C6—C5	1.4 (4)		

### Hydrogen-bond geometry (Å, °)

**Table:**

*D*—H···*A*	*D*—H	H···*A*	*D*···*A*	*D*—H···*A*
C6—H6···O1	0.94 (3)	2.22 (3)	2.833 (4)	121 (3)
N1—H1N···O2^i^	0.82 (3)	2.22 (3)	3.020 (3)	163 (3)

Symmetry codes: (i) −*x*+1, −*y*+1, −*z*.
